# NADPH Oxidase-Derived ROS Induced by Chronic Intermittent Hypoxia Mediates Hypersensitivity of Lung Vagal C Fibers in Rats

**DOI:** 10.3389/fphys.2016.00166

**Published:** 2016-05-09

**Authors:** Chang-Huan Yang, Wei-Ling Zhuang, Yan-Jhih Shen, Ching Jung Lai, Yu Ru Kou

**Affiliations:** ^1^School of Medicine, Institute of Physiology, National Yang-Ming UniversityTaipei, Taiwan; ^2^Department of Physiology, Tzu Chi UniversityHualien, Taiwan; ^3^PhD Program in Pharmacology and Toxicology, School of Medicine, Tzu Chi UniversityHualien, Taiwan

**Keywords:** chronic intermittent hypoxia, airway hypersensitivity, lung vagal C fibers, reactive oxygen species, NADPH oxidase

## Abstract

Obstructive sleep apnea (OSA), manifested by exposure to chronic intermittent hypoxia (CIH) and excess production of reactive oxygen species (ROS) in the airways, is associated with hyperreactive airway diseases. ROS, particularly when created by NADPH oxidase, are known to sensitize lung vagal C fibers (LVCFs), which may contribute to airway hypersensitivity pathogenesis. We investigated whether CIH augments the reflex and afferent responses of LVCFs to chemical stimulants and the roles of ROS and NADPH oxidase in such airway hypersensitivity. Rats were exposed to room air (RA) or CIH with/without daily treatment with MnTMPyP (a superoxide anion scavenger), apocynin (an NADPH oxidase inhibitor), or vehicle. At 16 h after their last exposure, intravenous capsaicin, adenosine, or α,β-methylene-ATP evoked an augmented apneic response in anesthetized rats with 14-days CIH exposure, compared to anesthetized rats with 14-days RA exposure. The augmented apneic responses to these LVCF stimulants were abolished by bilateral vagotomy or perivagal capsaicin treatment, which block LVCFs neural conduction and were significantly suppressed by treatment with MnTMPyP or apocynin, but not vehicle. Electrophysiological studies revealed that 14-days CIH exposure potentiated the responses of LVCFs to these stimulants. This effect was inhibited by treatment with MnTMPyP or apocynin treatment and was not seen in rats who received 7-days of CIH exposure. Biochemical analysis indicated that 14-days CIH exposure increased both lung lipid peroxidation, which is indicative of oxidative stress, and expression of the p47^phox^ subunit in the membrane fraction of lung tissue, which is an index of NADPH oxidase activation. The former was prevented by treatment with either MnTMPyP or apocynin, while the later was prevented by treatment with apocynin only. These results suggest that 14-days CIH exposure sensitizes LVCFs in rats, leading to an exaggerated reflex and afferent responses to stimulants and that this sensitization is mediated via ROS generated by NADPH oxidase.

## Introduction

Obstructive sleep apnea (OSA), manifested by airway exposure to chronic intermittent hypoxia (CIH), is associated with various hyperreactive airway diseases of humans including asthma (Teodorescu et al., 2012), chronic cough (Sundar et al., 2010), and bronchial hyperreactivity (Lin and Lin, 1995). These patients also display increased oxidative stress due to an excess production of reactive oxygen species (ROS) (Alonso-Fernandez et al., 2009; Hopps and Caimi, 2015). Indeed, in these patients, repetitive periods of upper airway collapse result in cyclic periods of hypoxia/re-oxygenation (Lavie, 2003), which seems to produce excess ROS in the airways (Prabhakar, 2001). The ROS derived from NADPH oxidase, a superoxide anion-generating enzyme, have been shown to contribute to several cardiorespiratory responses induced by CIH (Nisbet et al., 2009; Edge et al., 2012; Schulz et al., 2014). Upon activation, the cytosolic subunits of NADPH oxidase, including p47^phox^, are recruited to the plasma membrane and bind with the membrane subunits to form the active complex (Kou et al., 2011). While oxidative stress is implicated in the pathogenesis of several OSA-related diseases such as cardiovascular (Hopps and Caimi, 2015), neurodegenerative (Zhang and Veasey, 2012), and metabolic complications (Mesarwi et al., 2015), its role in the development of OSA-associated hyperreactive airway diseases is still not known.

Airway hypersensitivity is characterized by augmented sensory and reflex responses to stimuli due to sensitization of lung afferents by inflammatory mediators (Widdicombe, 2003; Kou et al., 2011). Particularly, stimulation of lung vagal C-fibers (LVCFs), a major type of lung afferents, triggers an array of defense reflex responses such as apnea, coughing, and bronchoconstriction (Coleridge and Coleridge, 1984; Lee and Pisarri, 2001). Thus, sensitization of LVCFs has been implicated in the pathogenesis of hyperreactive airway diseases (Widdicombe, 2003; Kou et al., 2011). Accumulating evidence suggests that excess ROS may sensitize C-fiber afferents, which then leads to the development of airway hypersensitivity (Tsai et al., 2006, 2009; Ruan et al., 2014). To this end, we recently reported (Shen et al., 2012) that 10 episodes [each consisting of 30 s of hypoxia air exposure followed by 30 s of room air (RA) exposure] of acute intermittent hypoxia in rats is able to induce LVCF-mediated airway hypersensitivity, and that this response can be prevented by pretreatment with antioxidants, which suggests the involvement of ROS. However, the experimental model in that study (Shen et al., 2012) is more akin to immediate hypersensitivity resulting from an acute exposure and this is not similar to the situation when CIH occurs in patients with OSA. Furthermore, different durations of intermittent hypoxia exposure are known to have different effects on respiration (Prabhakar and Kline, 2002). Accordingly, whether CIH may induce LVCF hypersensitivity remains to be investigated.

In the light of existing knowledge and the unanswered questions described above, this study was undertaken to investigate, firstly, whether exposure of rats to CIH for 14 days is able to augment the reflex and afferent responses of LVCFs to chemical stimulants and, secondly, whether ROS and NADPH oxidase play important roles in CIH-induced LVCF hypersensitivity if it occurs. To achieve these goals, anesthetized, spontaneously breathing rats and anesthetized, artificially ventilated rats were employed with the aim of studying the reflex and afferent responses, respectively. Responses to LVCF stimulants including capsaicin (Lin et al., 2013), adenosine (Lin et al., 2009), and α,β-methylene-ATP (Lin et al., 2013) were measured and these then served as indices of LVCF reactivity.

## Materials and methods

### Exposure to CIH

All experiments were performed on male adult Sprague-Dawley rats and the experimental procedures described below were approved by the Institutional Animal Care and Use Committee of the Tzu Chi University. Exposure of the animals to CIH was achieved using methods that have been described previously (Lai et al., 2006). Briefly, unrestrained, freely moving rats were housed in Plexiglas cylindrical chambers (length 28 cm, diameter 10 cm, volume 2.4 L) with snug-fitting lids. Pure nitrogen was allowed to enter the chamber for 30 s via a timed solenoid valve at a flow that was adjusted to reduce the ambient FIO_2_ to between 2 and 6% for 2–5 s. This was followed by infusion of compressed air for about 45 s, which allowed the gradual return of the ambient air to an FIO_2_ of 20.9%. The animals were exposed to CIH between 10:00 A.M. and 4:00 P.M. (6 h), 48 times per hour, for 7 or 14 consecutive days. The RA control group rats were exposed for 14 days to a similar pattern of gas dynamics in the chamber, but with the pure nitrogen being replaced with compressed air. At end of the exposure period, rats were removed from the exposure chambers and placed individually in clear acrylic chambers where they were cared regularly.

### Animal preparation

At 16 h after the last exposure to RA or CIH, the rats were anesthetized by giving them an intraperitoneal injection of α-chloralose (100 mg/kg; Sigma Chemical, St. Louis, MO) and urethane (500 mg/kg; Sigma) dissolved in a borax solution (2%; Sigma). The rats were tethered in a supine position and the neck opened along the midline; next a segment (~1 cm) of each vagus nerve was carefully isolated from the common carotid artery for later use. The trachea was then cannulated below the larynx using a short tracheal tube via a tracheotomy. A polyethylene catheter was inserted into the jugular vein and advanced until the tip was close to the right atrium; this was to allow the intravenous administration of pharmacological agents. The right femoral artery was cannulated in order to measure arterial blood pressure and heart rate. During the experiments, supplementary doses of α-chloralose (20 mg/kg/h) and urethane (100 mg/kg/h) were administered such that there were no pain reflexes on pinching the animal's tail. The body temperature of animals was maintained at about 36°C throughout the experiment by means of a servo-controlled heating blanket. The animals were sacrificed at the end of experiments by an intravenous injection of an overdose of the anesthetics.

### Measurements of ventilatory responses

For the reflex studies, rats were allowed to breath spontaneously via the tracheal cannula. Respiratory flow (VR) was measured using a pneumotachograph (Fleisch 4/0; Richmond, VA, USA) coupled with a differential pressure transducer (Validyne MP45-12), and was integrated to give tidal volume (VT). The tracheal pressure (Ptr) was monitored using a pressure transducer (Validyne MP45-28) via a side tap to the tracheal cannula.

### Perivagal capsaicin treatment

The technique used for perivagal capsaicin treatment was the same as a previous study and its aim was to selectively block the neural conduction of LVCFs (Kou et al., 1995). Briefly, cotton strips were soaked in capsaicin solution (250 μg/ml; Sigma) and these were then wrapped around a 2–3 mm segment of the isolated cervical vagus nerve. After 20 min, when the apneic reflex response to an intravenous injection of capsaicin (1 μg/kg) had been abolished, the cotton strips were removed. The blocking effect of perivagal capsaicin treatment on the reflex responses to capsaicin injection has been shown previously to last for 80–120 min (Kou et al., 1995).

### Recording of afferent activity

For the electrophysiological studies, afferent activities arising from the LVCFs were recorded in open-chest artificially ventilated rats as described in detail previously (Lai and Kou, 1998). Briefly, rats were ventilated with room air by a constant volume respirator (Harvard 683; South Natick, MA, USA); VT and frequency were set at 7–8 ml/kg and 60 breaths/min, respectively. A midline thoracotomy was performed and the expiratory outlet of the respiratory was placed under 3 cmH_2_O pressure to maintain a near-normal functional residual capacity. During the recording of the vagal action potentials, the rats were paralyzed with pancuronium bromide (0.05 mg/kg, i.v.; Orgnon Teknika, Boxtel, Holland). Periodically, the effect of pancuronium was allowed to wear off so that the depth of anesthesia could be checked. Briefly, a fine afferent filament was split from the desheathed nerve trunk of the right vagus and placed on a platinum-iridium recording electrode in order to record afferent nerve activity. The fine filament was further split until the afferent activity arising from a single unit could be detected. LVCF activity was searched for initially by examining the rat's mild response to lung hyperinflation (3–4 times VT) by occlusion of the expiratory line of the respirator. Furthermore, capsaicin (1 μg/kg), a potent chemical stimulant of LVCFs, was injected as a bolus into the right atrium. Only afferent fibers that showed stimulation within 2 s after the injection were studied. The conduction velocity of the afferent fibers of the receptors studied was measured by a method that has been described previously (Ho et al., 2001). Prior to the end of each experiment, the general locations of all fibers were identified by examining their responses to the gentle pressing of the lungs with a saline-wetted cotton Q-tip.

### Fluorescence assay for measuring lipid peroxidation in lung tissues

Lipid peroxidation was measured by a method that has been described previously (Kuo et al., 2011). After animals were sacrificed, lung tissue samples were quickly obtained and then stored at –80°C. In order to measure the lipid peroxidation present in the lung tissue samples, frozen lung tissue samples were thawed and homogenized in chilled 400 μl chloroform and 200 μl methanol by sonication. After centrifugation, an aliquot of the chloroform and methanol layer was scanned using a spectrofluorometer (Perkin-Elmer LS 50B; Waltham, MA, USA). The level of lipid peroxidation was determined by measuring malondialdehyde and its dihydropyridine polymers at an excitation wavelength of 356 nm and an emission wavelength of 426 nm.

### Western blot analysis for measuring the expression of NADPH oxidase subunit p47^phox^

Fresh lung tissue samples were obtained at 15 min after the last exposure to RA or CIH. To measure the expression of p47^phox^, the lung tissue samples were homogenized using a tissue grinder in permeabilization buffer that contained, phosphatase inhibitor (Thermo Fisher Scientific, USA), and protease inhibitor (iNtRON Biotechnology, Korea). In order to isolate membrane and cytoplasm proteins, a Mem-PERTM plus membrane protein extraction kit (Thermo) was used according to the manufacturer's instructions. Samples prepared using the kit were separated on 10% SDS-PAGE and then transferred to polyvinylidene fluoride membranes (Merck Millipore Corporation, USA). After blocking for 1 h with 5% non-fat milk at room temperature, the blots were incubated overnight at 4°C with goat primary antibody against rat p47^phox^ (1:2000; Abcam). After washed with Tris-buffered saline containing 0.1% Tween-20 three times, each for 5 min, the membranes were incubated with a second antibody, rabbit anti-goat IgG (1:1000; Sigma) for 1 h at room temperature; finally the protein bands were detected by enhanced chemiluminescence (GE Healthcare, USA). The signals were visualized by exposure of the membranes to X-ray film (Kodak, USA), and the relative signal intensities of the bands were quantified using ImageJ processing software.

### Pharmacological agents

To stimulate the LVCFs, capsaicin [1 μg/kg; a transient receptor potential vanilloid 1 (TRPV1) receptor agonist] (Lin et al., 2013), adenosine (an adenosine A1-receptor agonist; 200 μg/kg, Sigma) (Lin et al., 2009), and α,β-methylene-ATP (15 μg/kg in the reflex studies and 100 μg/kg in the electrophysiological studies; a P2X receptor agonist) (Lin et al., 2013) were injected as a bolus into the right atrium (volume 0.1 ml) and then flushed by an injection of 0.3 ml of saline. MnTMPyP [manganese (III) tetrakis(1-methyl-4-pyridyl)porphyrin; a potent scavenger of superoxide anions; 5 mg/kg/day; Calbiochem, San Diego, CA] and apocynin (an inhibitor of NADPH oxidase; 30 mg/kg/day, Sigma) were given daily by intraperitoneal injection (volume ~0.35 ml) at 15 min prior to CIH or RA exposure for 14 consecutive days. The vehicle for adenosine, α,β-methylene-ATP, and MnTMPyP was saline. The vehicle for capsaicin was a solution that contained 10% Tween 80, 10% ethanol, and 80% saline. Apocynin was prepared by dissolving the agent in 20% dimethyl sulfoxide (Sigma) and then diluting the resulting solution with saline.

### Experimental design and protocols

A total of 152 rats (weight 320–445 g) were used in this study. During the reflex and electrophysiological studies, 140 rats were divided into 14 study groups to allow four series of experiments to be performed; each group consisted of 10 rats and only one LVCF was studied in each rat as part of the electrophysiological studies. The injections of the three types of chemical stimulants (capsaicin, adenosine, and α,β-methylene-ATP) were performed separately in a counterbalance order. To allow the baseline respiratory pattern or fiber activity (FA) to return to its control level, an elapsed time of ~15 min was allowed between any two injections of stimulants. In study series 1, the apneic reflex responses to the three stimulants were studied in rats exposed to RA (*Group 1*) or CIH for 14 days (*Group 2*), with the aim being to study the potentiating effect of CIH. Subsequently, the apneic responses to the three stimulants were measured after perivagal capsaicin treatment and then after a bilateral cervical vagotomy to assess the role of the LVCFs. In study series 2, the afferent responses of LVCFs to the three stimulants were evaluated in rats exposed to RA for 14 days (*Group 3*), CIH for 7 days (*Group 4*), and CIH for 14 days (*Group 5*) with the aim of assessing the time-dependent effect of CIH. In study series 3, the apneic reflex responses and afferent responses of the LVCFs to the three stimulants were investigated in rats exposed to RA or CIH for 14 days. Both 14-days RA and 14-days CIH rats received daily treatment with MnTMPyP (Mn + CIH and Mn+RA; *Groups 6-8*), apocynin (Apo + CIH and Apo + RA; *Groups 9-11*), and their vehicle (Veh + CIH and Veh + RA; *Groups 12-14*), with the aim being to assess the roles of superoxide anion and NADPH oxidase in the responses to CIH. In study series 4, lung tissue samples were obtained from *Groups 1, 2, 6, 9*, and *12* where the rats had been exposed to RA or CIH for 14 days in the reflex studies; these were employed to measure the level of lipid peroxidation in the various lung tissue sample with the aim of assessing the CIH-induced oxidative stress. In the part of the study involving the activation of NADPH oxidase by CIH, lung tissue samples were obtained from three additional groups of rats (each *n* = 4) that had been exposed to RA, to CIH, and to CIH with daily apocynin treatment; this was to allow measurement of the protein levels of p47^phox^.

### Data analysis and statistics

As part of the studies of the reflex responses, respiratory frequency (*f*), expiratory duration (TE), and VT were analyzed on a breath-by-breath basis as the average value over the 10-breath period immediately before injection of chemical stimulants. To compare the apneic responses evoked by different experimental conditions, the longest TE occurring during the first 20 s after injection of the stimulant was divided by the baseline TE to yield the apneic ratio. For the studies of LVCF responses, baseline FA was calculated as the average value over a 10-s interval immediately preceding injection of the chemical stimulants. The peak responses were defined as the maximum averaged over a 2-s interval during the 20 s following the injection of the stimulant. In all studies, mean arterial blood pressure and heart rate were continuously analyzed at 1-s intervals. Baseline arterial blood pressure (ABP) and heart rate (HR) were calculated as the mean value over the 10-s period immediately preceding injection of the stimulant. All physiological signals were analyzed by a computer equipped with an analog-to-digital converter (Gould DASA 4600) and appropriate software (BioCybernatics 1.0, Taipei, Taiwan). Data obtained from three or more groups were compared by one-way ANOVA or two-way mixed factorial ANOVA, followed by Neuman–Keuls tests when appropriate. A value of *p* < 0.05 was considered significant. All data are presented as mean ± SE.

## Results

### Baseline physiological parameters

There was no significant difference in mean body weight between the 14-days RA rats and the 14-days CIH rats with or without daily drug treatments either at the beginning of exposure or at termination of exposure (Table 1). Among animals without any daily treatment with drugs, the mean ABP (128.85 ± 2.12 mmHg), but not the HR (342.52 ± 10.93 beats/min), of the 14-days CIH rats (*n* = 20) was significantly greater than that of the 14-days RA rats (ABP = 107.06 ± 3.03 mmHg; HR = 330.80 ± 10.03 beats/min) (*n* = 20) when anesthetized. Furthermore, during the reflex studies, the baseline f (75.09 ± 1.73 breaths/min), TE (0.51 ± 0.02 s), and VT (1.87 ± 0.10 ml) of the 14-days CIH rats (*n* = 10) were similar to those of the 14-days RA rats (*f* = 70.53 ± 2.02 breaths/min; TE = 0.55 ± 0.02 s; VT = 1.99 ± 0.10 ml) (*n* = 10). In the electrophysiological studies, a total of 90 LVCF fibers were measured the responses to chemical stimulants. The baseline LVCF activities of the rats exposed to 7- or 14-days CIH without daily drug treatment were not significantly different from that of the rats exposed to 14-days RA without daily drug treatment (Table 2). The mean baseline LVCF activity was also not significantly altered by administration of MnTMPyP and apocynin, as compared with that of treatment with vehicle in either 14-days RA rats or 14-days CIH rats (Table 2). Of the 90 LVCFs studied, the conduction velocity of 76 fibers was measured (1.08 ± 0.06 m/s; range 0.74–1.77 m/s). The conduction velocity of remaining 14 LVCFs was not measured due to loss of electrophysiological signal. These LVCFs were all localized within the lung structure.

**Table 1:** **Baseline body weight at the beginning and termination of 14-days room air (RA) exposure or 14-days chronic intermittent hypoxia (CIH) exposure in rats**.

	**Body weight (g)**					
	**RA for 14 days**			**CIH for 14 days**		
	***N***	**At beginning**	**At termination**	***N***	**At beginning**	**At termination**
No treatment	24	343.5 ± 11.7	390.8 ± 17.6	24	339.5 ± 13.2	372.1 ± 16.8
Mn treatment	10	335.7 ± 13.6	389.0 ± 19.6	20	340.6 ± 15.3	379.5 ± 17.4
Apo treatment	10	338.1 ± 12.8	394.0 ± 18.4	24	330.4 ± 14.1	375.4 ± 16.5
Veh treatment	10	336.5 ± 14.1	391.5 ± 20.6	20	329.3 ± 15.9	370.8 ± 18.3

*Body weights were measured at the beginning of exposure and at the termination of exposure. Rats were exposed to 14-days RA or 14-days CIH with or without daily treatment with MnTMPyP (Mn treatment), apocynin (Apo treatment) or their vehicle (Veh treatment). Data in each group are the means ± SE. N, animal numbers. There was no significant difference (P > 0.05) in mean body weight between the 14-days RA rats and the 14-days CIH rats with or without daily drug treatments either at the beginning of exposure or at the termination of exposure*.

**Table 2:** **Baseline fiber activity of lung vagal C fibers (LVCFs) in various study groups**.

**Group**	**Baseline activity (impulses/s)**			
	**No treatment**	**Mn treatment**	**Apo treatment**	**Veh treatment**
RA 14	0.04 ± 0.02	0.03 ± 0.02	0.03 ± 0.02	0.04 ± 0.02
CIH 14	0.10 ± 0.03	0.04 ± 0.01	0.03 ± 0.02	0.09 ± 0.02
CIH 7	0.06 ± 0.02	–	–	–

*Rats were exposed to 14-days room air (RA 14), 14-days chronic intermittent hypoxia (CIH 14), or 7-days CIH (CIH 7) with or without daily treatment with MnTMPyP (Mn treatment), apocynin (Apo treatment) or their vehicle (Veh treatment). Data in each group (means ± SE; n = 10) are values averaged over 10-s periods immediately preceding the capsaicin injection in the electrophysiological studies. Only one LVCF fiber was studied in each rat. No statistical significance between any two groups was detected (P > 0.05)*.

### Role of LVCFs in the CIH-induced augmented apneic response to chemical stimulants

**Figure 1:**
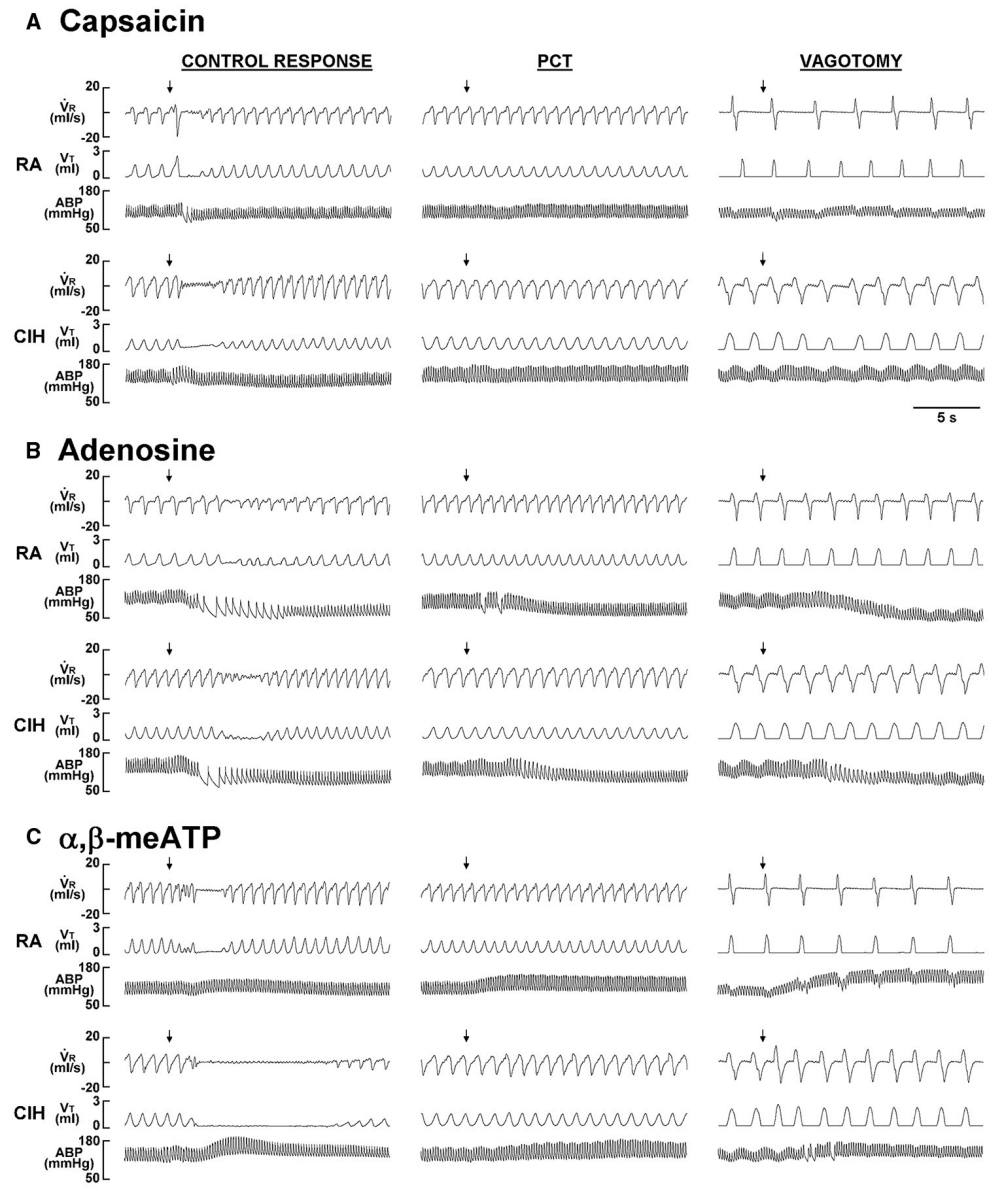
**Ventilatory responses to the intravenous injection of three types of stimulants in two rats after exposure to room air or chronic intermittent hypoxia**. Duration of exposure to room air (RA) or chronic intermittent hypoxia (CIH) was 14 days. At 16 h after the last exposure, the animals' responses to capsaicin (1.0 μg/kg; **A**), adenosine (0.2 mg/kg; **B**), and α,β-methylene-ATP (α,β-meATP; 15 μg/kg; **C**) were measured in each rat under control conditions, after perivagal capsaicin treatment (PCT; 250 μg/ml), and then after bilateral cervical vagotomy. These drugs are stimulants of lung vagal C fibers and were injected into the jugular vein as a bolus (0.1 ml volume) as indicated by the arrows. The injection catheter had its tip close to the right atrium. Approximately 15 min elapsed between any two injections. VR, respiratory flow; VT, tidal volume; ABP, arterial blood pressure.

**Figure 2:**
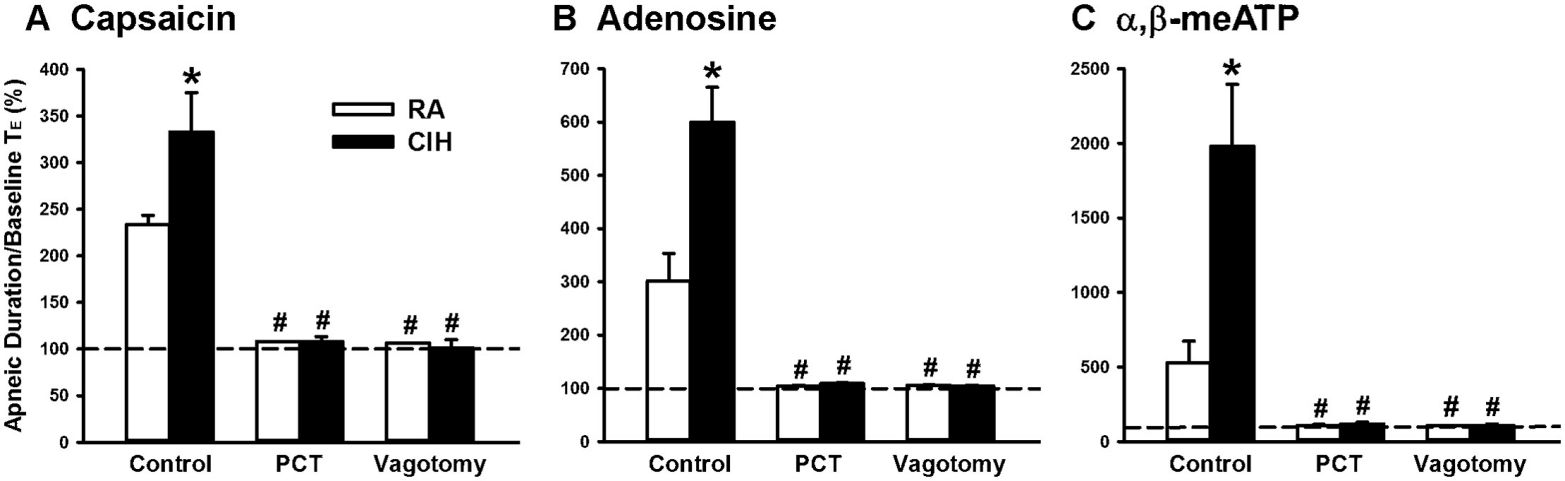
**Role of lung vagal C fibers in the chronic intermittent hypoxia-induced augmented apneic responses to stimulants in rats**. Apneic responses to intravenous capsaicin **(A)**, adenosine **(B)**, and α,β-meATP **(C)** were measured in a counterbalance order 16 h after 14-days exposure to room air (RA) or chronic intermittent hypoxia (CIH) in two groups of rats. For each rat, the responses to these stimulants were measured under control conditions, after perivagal capsaicin treatment (PCT), and then after vagotomy. The longest expiratory duration (TE) occurring during the first 20 s after stimulant injection was divided by the baseline TE to yield the apneic ratio. Baseline TE was calculated as the average over 10 consecutive breaths immediately before injection. The horizontal dashed lines indicate an apneic ratio of 100% (no response). ^*^p < 0.05 compared with responses in the RA group under the same experimental condition; ^#^*p* < 0.05 compared with control responses in the same group. Data in each group are means ± SE of 10 rats. See the legend of Figure 1 for further explanation.

When the 14-days RA rats were investigated, intravenous capsaicin was found to induce a mild inhibitory effect on breathing, which in turn led to apnea appearing as a prolonged TE (Figure 1A). Interestingly, the prolonged TE evoked by the same dose of capsaicin was greatly augmented among the 14-days CIH rats (Figure 1A). As a group, the average apneic response to capsaicin among the 14-days CIH rats was significantly greater than that of the 14-days RA rats (Figure 2A). The potentiating effect of CIH was not limited to the apneic response caused by capsaicin. Similarly, the apneic responses to intravenous adenosine (Figures 1B, 2B) and α,β-methylene-ATP (Figures 1C, 2C) were significantly augmented among the 14-days CIH rats compared with the 14-days RA rats. Further analysis revealed that perivagal capsaicin treatment or vagotomy, two procedures that block the neural conduction of LVCFs (Lin et al., 2013), totally abolished the apneic responses to intravenous capsaicin, adenosine, and α,β-methylene-ATP in both 14-days RA and 14-days CIH rats (Figures 1, 2).

### The potentiating effect of CIH on the LVCF responses to chemical stimulants

**Figure 3:**
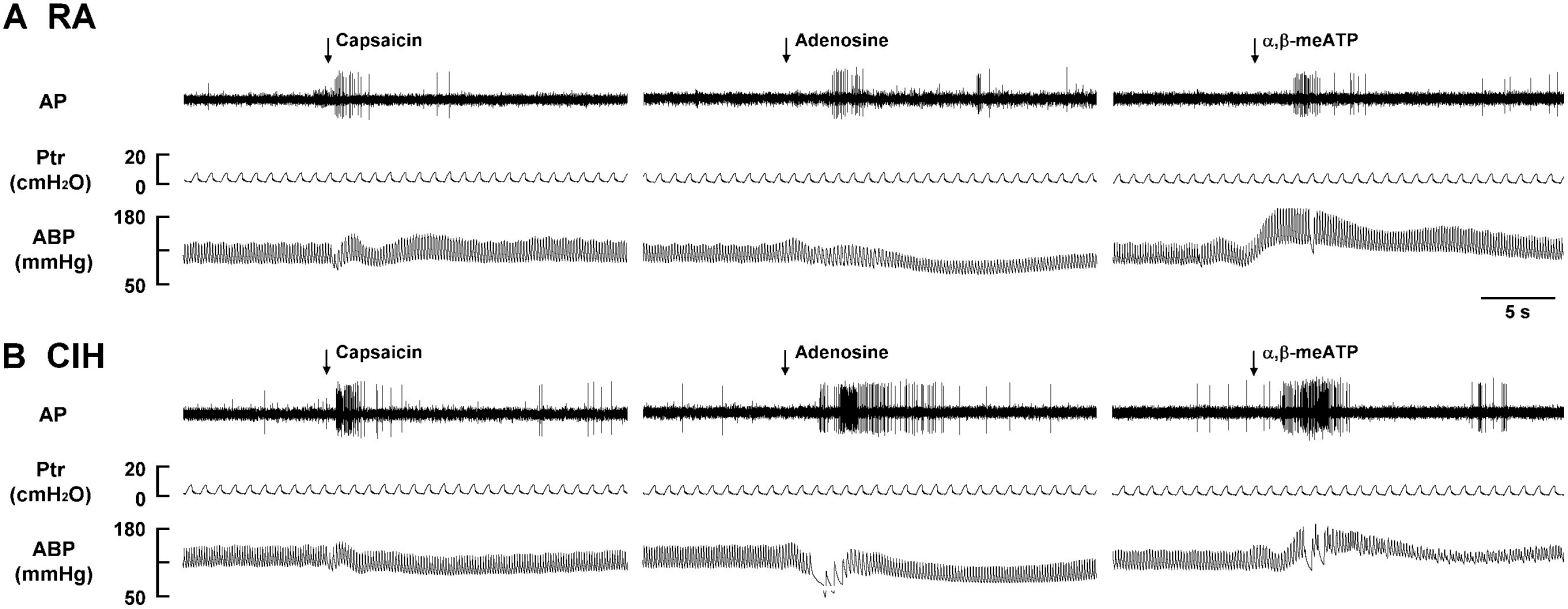
**Responses of lung vagal C fibers to intravenous injection of three types of stimulants in two rats after exposure to room air or chronic intermittent hypoxia**. Duration of exposure to room air (RA; **A**) or chronic intermittent hypoxia (CIH; **B**) was 14 days. At 16 h after the last exposure, afferent responses to intravenous capsaicin, adenosine, and α,β-meATP were measured. These stimulants were injected into the jugular vein as a bolus (0.1 ml volume) as indicated by the arrows. Approximately 15 min elapsed between any two injections. AP, action potential; Ptr, tracheal pressure; ABP, arterial blood pressure. See the legend of Figure 1 for further explanation.

**Figure 4:**
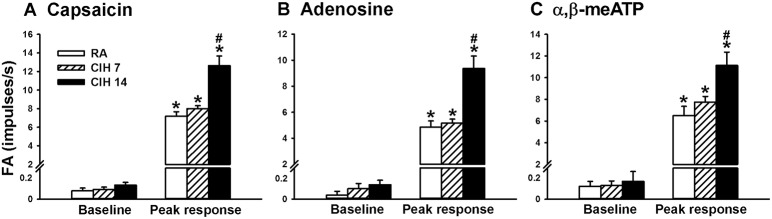
**The sensitizing effect of chronic intermittent hypoxia on afferent responses of lung vagal C fibers to intravenous stimulants in rats**. Three groups of rats received exposure to room air (RA) for 14 days, chronic intermittent hypoxia for 7 days (CIH 7) or CIH for 14 days (CIH 14). Sixteen hours after the last exposure, the responses to intravenous capsaicin **(A)**, adenosine **(B)**, and α,β-meATP **(C)** were measured in a counterbalance order. Baseline FA was calculated as the value averaged over 10-s interval before stimulation. The peak response was measured as the maximum averaged over 2-s interval after stimulation. Data in each group are means ± SE of 10 fibers recorded from 10 rats. ^*^p < 0.05 compared with baseline in the same group; ^#^*p* < 0.05 compared with the peak response in the RA group. See the legend of Figure 1 for further explanation.

Since, the LVCFs were found to mediate the CIH-induced augmented apneic responses as outlined above, we next measured the activity of these afferents when evoked by chemical stimulation. In the 14-days RA rats (Figure 3A), intravenous capsaicin, adenosine, or α,β-methylene-ATP all evoked an abrupt discharge burst. The discharge evoked by any one of these stimulants was markedly augmented among the 14-days CIH rats (Figure 3B). As a group, there was no significant difference in the baseline activities of the LVCFs between the 14-days RA rats and the 14-days CIH rats (Figure 4). However, the average peak responses of the LVCFs to capsaicin (Figure 4A), to adenosine (Figure 4B), and to α,β-methylene-ATP (Figure 4C) among the 14-days CIH rats were all significantly greater than those of the 14-days RA rats. By way of contrast, this potentiating effect of CIH on the LVCF responses to chemical stimulants was not found among rats that had been exposed to CIH for only 7 days (Figure 4).

### Roles of ROS and NADPH oxidase in the potentiating effect of CIH

**Figure 5:**
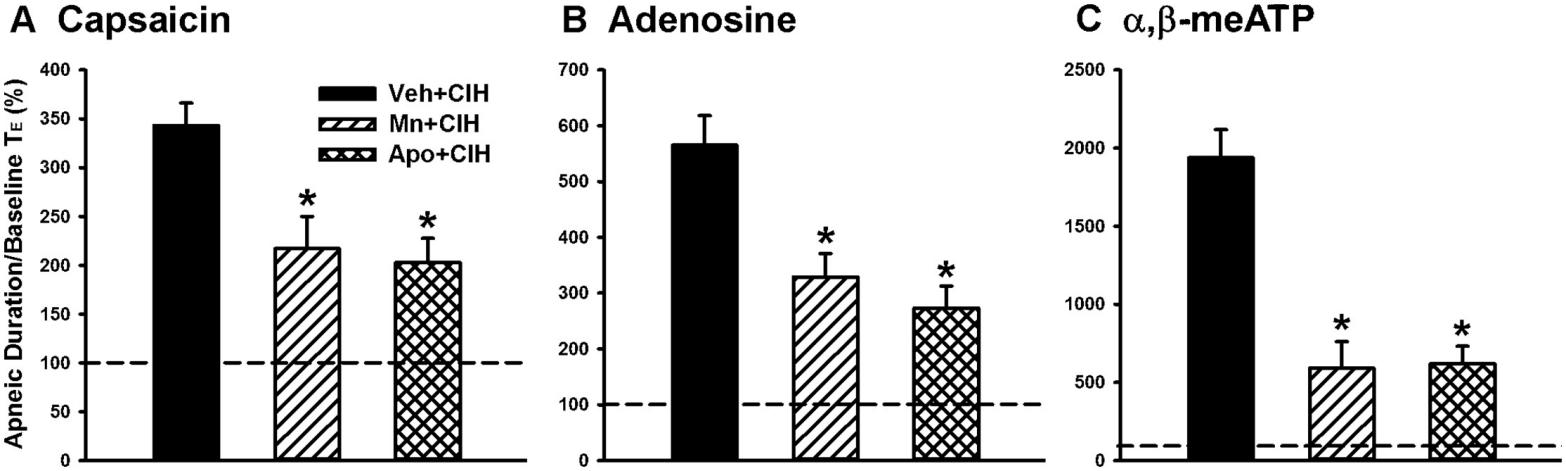
**Roles of reactive oxygen species and NADPH oxidase in the chronic intermittent hypoxia-induced potentiating effect on apneic responses to stimulants in rats**. Apneic responses to intravenous capsaicin **(A)**, adenosine **(B)**, and α,β-meATP **(C)** were measured in a counterbalance order from each rat at 16 h after 14-days exposure to chronic intermittent hypoxia (CIH) in three groups of rats. Each group of rats received daily treatment with MnTMPyP (a superoxide anion scavenger, 5 mg/kg/day, i.p.; Mn + CIH), apocynin (an NADPH oxidase inhibitor, 30 mg/kg/day, i.p.; Apo + CIH), or their vehicle (i.p.; Veh + CIH). The horizontal dashed lines indicate an apneic ratio of 100% (no response) ^*^p < 0.05 compared with Veh+CIH in each panel. Data in each group are the means ± SE of 10 rats. See the legends of Figures 1, 2 for further explanation.

**Figure 6:**
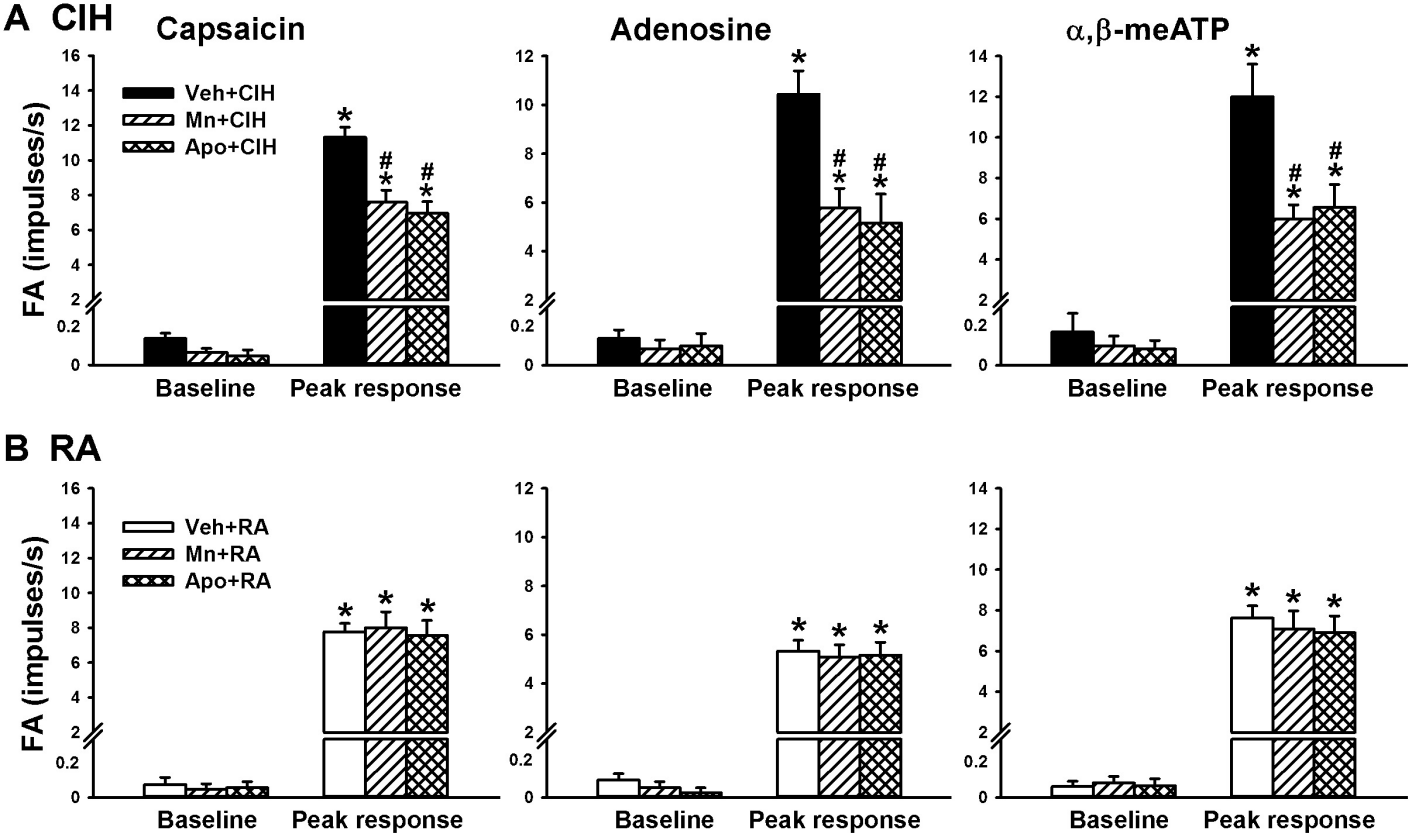
**Roles of reactive oxygen species and NADPH oxidase in the chronic intermittent hypoxia-induced potentiating effect on afferent responses of lung vagal C fibers to intravenous stimulants in rats**. Afferent responses (fiber activity, FA) to intravenous capsaicin, adenosine or α,β-meATP were measured in a counterbalance order from each rat 16 h after 14-days exposure to chronic intermittent hypoxia (CIH; **A**) or room air (RA; **B**) in three groups of rats. Each group of 14-days CIH rats and 14-days RA rats received daily treatment with MnTMPyP (Mn + CIH; Mn + RA), apocynin (Apo + CIH; Apo + RA), or their vehicle (Veh + CIH; Veh + RA). Data in each group are the means ± SE of 10 fibers recorded from 10 rats. ^*^p < 0.05 compared with baseline in the same group; ^#^*p* < 0.05 compared with peak response in the Veh + CIH group. See the legends of Figures 1, 3 for further explanation.

Since ROS and NADPH oxidase contribute to various other cardiorespiratory consequences induced by CIH (Nisbet et al., 2009; Edge et al., 2012; Schulz et al., 2014), we employed a superoxide anion scavenger (MnTMPyP) and an inhibitor of NADPH oxidase (apocynin) to assess their roles in the potentiating effect of CIH. In the reflex studies, the augmented apneic responses to capsaicin (Figure 5A), adenosine (Figure 5B), and α,β-methylene-ATP (Figure 5C) observed among the 14-days CIH rats with vehicle treatment were found to be significantly reduced in the 14-days CIH rats after treatment with either MnTMPyP or apocynin. Consistently, in the electrophysiological studies, the augmented peak responses of LVCFs to capsaicin, adenosine, and α,β-methylene-ATP) that were observed among the 14-days CIH rats after vehicle treatment were also found to be significantly attenuated among the 14-days CIH rats after treatment with either MnTMPyP or apocynin (Figure 6A). By way of contrast, treatment with either MnTMPyP or apocynin did not significantly affect the peak responses of LVCFs to these chemical stimulants among the 14-days RA rats (Figure 6B).

### Levels of lipid peroxidation and of p47^phox^ expression in rat lung tissue

**Figure 7:**
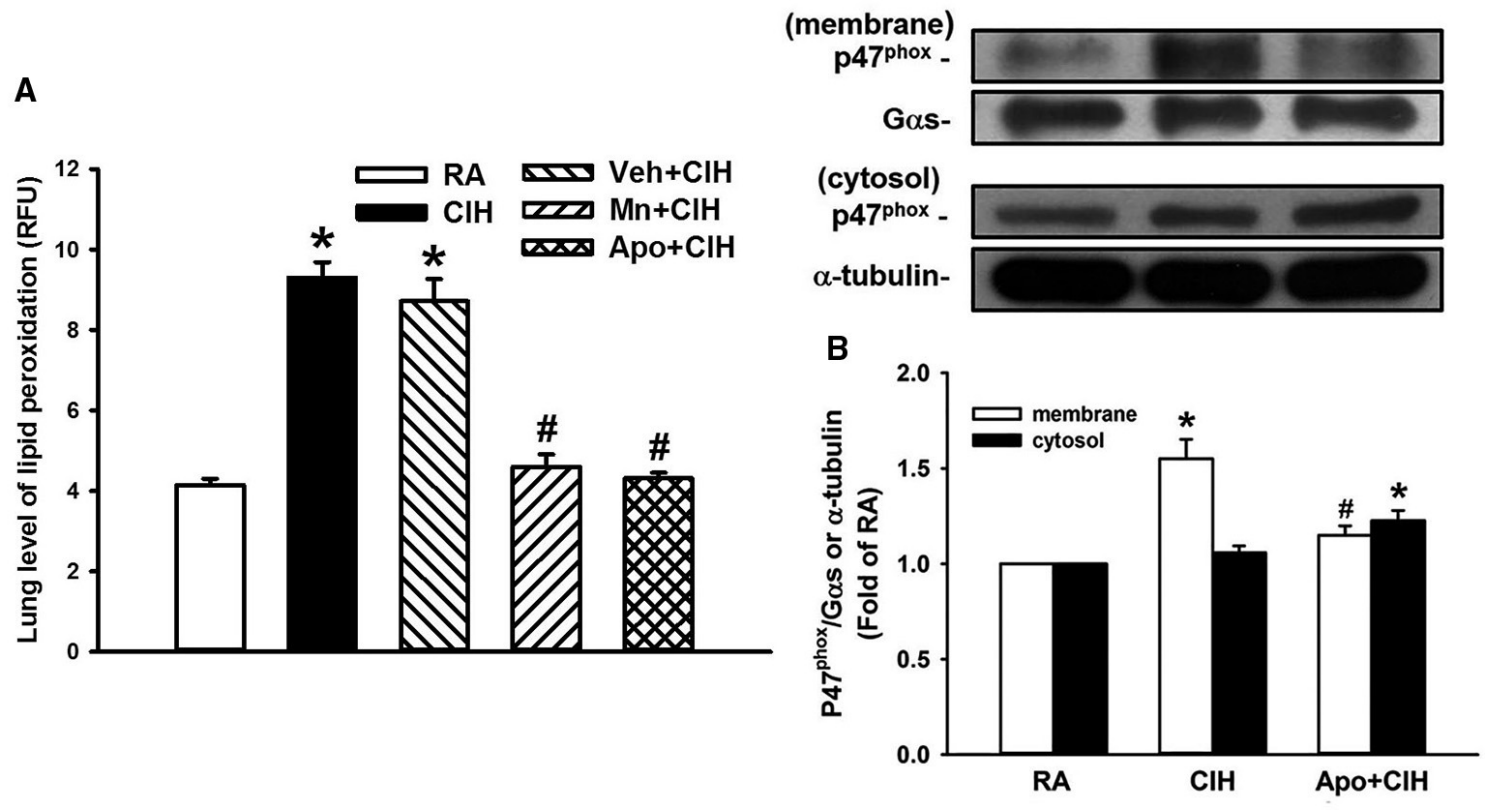
**Levels of lipid peroxidation and expression of p47^phox^ in rat lung tissue samples**. In **(A)**, two groups of rats were exposed to room air (RA) or chronic intermittent hypoxia (CIH) for 14 days. The other three groups were exposed to CIH for 14 days with daily treatment with MnTMPyP (Mn+CIH), apocynin (Apo+CIH) or their vehicle (Veh+CIH). The levels of lipid peroxidation in lung tissue samples were measured by fluorescence assay in order to assess the level of oxidative stress. RFU, relative fluorescence unit. In **(B)**, additional rats were exposed to RA or CIH for 14 days with or without daily apocynin treatment. The levels of the protein expression of p47^phox^ in the membrane and cytosolic fractions of lung tissue samples were analyzed by Western blotting to assess the activation of NADPH oxidase. Data in each group in **(A,B)** are means ± SE of 10 and 4 rats, respectively. In **(A)**, ^*^p < 0.05 compared with the level in the RA group; #*p* < 0.05 compared with the level in the Veh+CIH group. In **(B)**, ^*^p < 0.05 compared with the level of the same fraction in the RA group; #*p* < 0.05 compared with the level of the same fraction in the CIH group.

Since ROS and NADPH oxidase play important roles in the potentiating effect of CIH, we measured levels of lung lipid peroxidation and protein expression of p47^phox^ in the membrane and cytosolic fractions of lung tissue samples; these results should reflect the level of oxidative stress and the activation of NADPH oxidase in these samples. Indeed, the level of lipid peroxidation in the lung tissue samples from the 14-days CIH rats without any treatment was significantly greater than that of the samples from the 14-days RA rats (Figure 7A). This CIH-induced increase in lung lipid peroxidation was not seen among the 14-days CIH rats with daily treatment with either MnTMPyP or apocynin, but was unaffected by treatment with vehicle (Figure 7A). Additionally, the presence of p47^phox^ in the membrane fraction was found to be significantly increased among the 14-days CIH rats as compared to that among the 14-days RA rats (Figure 7B). This CIH-induced increase in the expression of membrane p47^phox^ was totally blunted among the 14-days CIH rats that had undergone daily treatment with apocynin (Figure 7B).

## Discussion

The results of this study demonstrate that, 16 h after the last exposure, rats treated with 14-days CIH exposure displayed augmented apneic responses to an intravenous injection of three different LVCF stimulants (capsaicin, adenosine, and α,β-methylene-ATP) as compared to rats treated with 14-days RA exposure (Figures 1, 2). The apneic responses to these stimulants were abolished by perivagal capsaicin treatment or vagotomy (Figures 1, 2), which suggests the responses are consequential reflexes that are mediated via the LVCFs. Indeed, our electrophysiological studies revealed that 14-days CIH exposure also potentiated the LVCF responses to these stimulants (Figures 3, 4). The potentiating effect of 14-days CIH on the apneic responses (Figure 5) or the LVCF responses (Figure 6A) to these stimulants were largely attenuated by daily treatment with either MnTMPyP (a superoxide anion scavenger) or apocynin (an inhibitor of NADPH oxidase), which indicates the involvements of ROS and NADPH oxidase in these processes. The suppressive effects of these two drugs are unlikely due to any deleterious influence on LVCFs because they did not affect the LVCF responses to stimulants when rats treated with 14-days RA exposure were investigated (Figure 6B). Collectively, these results suggest that 14-days CIH exposure sensitizes the LVCFs and that this leads to both an augmented reflex response and an augmented afferent response in rats to the above stimulants and this sensitization requires the participation of ROS and NADPH oxidase.

Since, both ROS and NADPH oxidase are important contributors to the development of CIH-induced sensitization of LVCFs, it is conceivable that they are closely linked. In fact, concomitant with the sensitization of LVCFs, 14-days CIH exposure also induced an increase in the lung level of lipid peroxidation, which could be prevented by treatment with MnTMPyP or apocynin (Figure 7A). These findings indicate that CIH exposure may promote the production of ROS via activation of NADPH oxidase. This notion is further supported by our finding that CIH exposure increased the presence of p47^phox^ in the membrane fraction of lung tissue samples and that this was totally blunted by treatment with apocynin (Figure 7B). In this case it would seem that the elevated expression of membrane p47^phox^ reflects the increased translocation of cytosolic p47^phox^ to the plasma membrane, which is a crucial step in the activation of NADPH oxidase (Kou et al., 2011). CIH exposure involves cyclic periods of hypoxia/re-oxygenation in the airways (Prabhakar, 2001) and it is well-documented that hypoxia (Mittal et al., 2012) and re-oxygenation (Abramov et al., 2007) each may independently evoke an increase in NADPH oxidase activity. Although activation of NADPH oxidase is known to be a major source of CIH-induced ROS generation (Prabhakar, 2011), we cannot completely rule out other additional sources of ROS, such as the mitochodria and/or xanthine oxidase. Our observations regarding the importance of ROS derived from NADPH oxidase are consistent with those in a study of CIH-induced cardiorespiratory changes including the increased occurrence of apnea during sleep in rats (Edge et al., 2012), augmentation of the chemoreflex control of sympathetic activity in rats (Marcus et al., 2010), pulmonary hypertension in mice (Nisbet et al., 2009), sensory plasticity of the carotid body in rats and mice (Peng et al., 2009), and arterial hypertension in mice (Schulz et al., 2014).

The mechanism by which CIH exposure dramatically enhances the sensitivity of LVCFs to different chemical stimulants, which, in turn, leads to exaggerated reflex responses, remains unclear. Recent studies have reported that excess ROS may sensitize C-fiber afferents, which in turn leads to the development of airway hypersensitivity (Tsai et al., 2006, 2009; Shen et al., 2012; Ruan et al., 2014) and thus ROS are likely to be the major mediators responsible for the CIH-induced consequences observed in this study. Additionally, excessive ROS may promote the release of other mediators such as cyclooxygenase metabolites and ATP (Chakraborti et al., 1989; Matyas et al., 2002; Tsai et al., 2009) within the airways, many of which are also able to sensitize airway C-fiber afferents (Ho et al., 2000; Gu et al., 2003; Tsai et al., 2009). Capsaicin, adenosine, and α,β-methylene-ATP are agonists of TRPV1, adenosine A1, and P2X receptors, respectively, which are located at the terminals of the LVCFs (Lin et al., 2009, 2013). The TRPV1 receptors and P2X receptors are non-selective cationic channels that are mainly permeable to Ca^2+^ (Taylor-Clark and Undem, 2006, 2011), whereas the adenosine A1 receptors are G protein-coupled metabotropic receptors (Taylor-Clark and Undem, 2006). Thus, it appears that the CIH-induced ROS and perhaps other potential mediators are able to produce non-specific increases in the electrical excitability of the LVCFs regardless of the stimulation by different types of agonists. Alternatively, activation of these three types of receptors by the CIH-induced ROS and other potential mediators may possibly be promoted by a common cellular mechanism that sensitizes the functioning of both ionotropic and metabotropic receptors. Indeed, these three types of receptors are known to mediate the sensitization of airway C-fiber afferents induced by other experimental interventions (Gu et al., 2003; Tsai et al., 2006, 2009; Ruan et al., 2014). Whatever the mechanism, the CIH exposure appears to only increase the sensitivity of LVCFs to chemical stimulants because the baseline activity of these afferents did not significantly change after 14-days CIH exposure.

Several clinical studies have reported that patients with OSA show an association with asthma (Teodorescu et al., 2012), chronic cough (Sundar et al., 2010), and bronchial hyperreactivity (Lin and Lin, 1995). LVCF hypersensitivity has been implicated in the development of these hyperreactive airway diseases (Lee and Pisarri, 2001; Gu et al., 2003; Taylor-Clark and Undem, 2011). More impotantly, nasal continuous positive airway pressure (CPAP) has been shown to reduce the symtoms of asthma (Ciftci et al., 2005; Teodorescu et al., 2012), chronic cough (Sundar et al., 2010), and bronchial hyperreactivity (Lin and Lin, 1995) in patients with OSA. CPAP is a standard therapy to alleviate repetitive episodes of hypoxia/re-oxygenation and has also been found to be effective at preventing ROS production in these patients (Alonso-Fernandez et al., 2009). These clinical studies highlight the link between excess ROS and hyperreactive airway diseases in this patient population. In this context, we have previously reported (Shen et al., 2012) that 10 episodes of acute intermittent hypoxia is able to potentiate the sensitivity of LVCFs to chemical stimulants and that this sensitizing effect is also mediated through the action of ROS. Actually, the sensitizing effect observed in the acute IH exposure model was immediately detectable as early as 10 min after termination of exposure; however, the time for the disappearance of the sensitizing effect was not determined in that study (Shen et al., 2012). In this study, we observed the hypersensitivity of LVCFs to the three chemical stimulants at 16 h after the termination of 14-days CIH exposure, which suggests that the sensitizing effect is very persistent. Contrastingly, this sensitizing effect of CIH was not seen at 16 h after the termination of 7-days CIH exposure (Figure 4). This finding is in good agreement with the hypothesis that the duration of exposure is important for the effect of intermittent hypoxia on respiration (Prabhakar and Kline, 2002). Perhaps, the experimental model in this study is more similar to the situation when CIH occurs in patients with OSA.

In conclusion, the results from the present study indicate that 14-days CIH exposure sensitizes LVCFs, which leads to an exaggerated reflex and afferent response to stimulants in rats and this sensitization is mediated through the action of ROS derived from NADPH oxidase. Our findings seem to provide support for the beneficial effect of antioxidants as a potential therapeutic strategy when preventing the hyperreactive airway diseases that are associated with OSA.

## Author contributions

The conception and design of the research: CJL and YRK. Performance of the experiments: CHY, WLZ, and YJS. Analysis and interpretation of the data: CHY, WLZ, YJS, CJL, and YRK. Preparation of the figures: CHY, WLZ, YJS, CJL, and YRK. Edited and revision of the manuscript: CHY, WLZ, YJS, CJL, and YRK.

## Funding

This study was supported by grants MOST 104-2320-B-010-014-MY3 and NSC 98-2628-B-320-003-MY3 from Ministry of Science and Technology, Taiwan, and a grant TCIRP101003-01 from Tzu Chi University, Taiwan.

### Conflict of interest statement

The authors declare that the research was conducted in the absence of any commercial or financial relationships that could be construed as a potential conflict of interest.
